# Awareness, perceptions, and clinical use of deep margin elevation in subgingival restorative cases: A cross-sectional multicentric study

**DOI:** 10.1371/journal.pone.0341933

**Published:** 2026-07-24

**Authors:** Jitendra Jethwani, Gayathri Sundari, Lovely M. Annamma, Esam Tashkandi, Caroline K. Carrico

**Affiliations:** 1 School of Dentistry, Virginia Commonwealth University, Richmond, Virginia, United States of America; 2 Department of Restorative Dentistry, Saveetha Dental College, Chennai, Tamil Nadu, India; 3 Dental Research Cell, Dr D. Y. Patil Dental College and Hospital, Dr D. Y. Patil Vidyapeeth, Pune, Maharashtra, India; 4 Department of Prosthodontics, College of Dentistry, King Saud University, Riyadh, Saudi Arabia; Danube Private University, AUSTRIA

## Abstract

**Background:**

Deep margin elevation (DME) has been introduced as a restorative approach to manage subgingival margins and facilitate adhesive procedures. Despite its clinical description in the literature, limited information is available regarding dentists’ awareness of the technique and their willingness to incorporate it into practice.

**Objective:**

To assess awareness, perceptions and clinical use of DME for subgingival restorative cases among practicing dentists from selected regions.

**Methodology:**

A cross-sectional, questionnaire-based study was conducted among dentists working in academic institutions, private practices, or combined settings across centers in the Middle East, the United States, Asia (India), Europe (UK) and practicing dentists in the fellows of the Academy of Dentistry International, stated as other regions. A 20-item closed-ended, self-administered questionnaire assessed awareness and clinical acceptance of DME. Data were analyzed using descriptive statistics and the Chi-square test using SAS (SAS Institute, Cary, NC), with significance set at 0.05.

**Results:**

Of 450 invited dentists, 349 completed the survey (response rate: 77.6%). Participants practiced in academic institutions (23%), private settings (39%), or combined academic–private environments (38%). Most respondents (77%) were familiar with DME, and 66% considered adhesive bonding to cervical or root dentin in deep margin situations to be predictable. However, when favorable clinical conditions were present, 75% reported preferring surgical crown lengthening. Reported routine use of DME for extensive subgingival proximal lesions in posterior teeth was limited.

**Conclusion:**

Most respondents were aware of deep marginal elevation (DME) as a successful approach for improving tooth longevity and restoration success when managing deep subgingival margins, provided proper isolation is maintained during the procedure. Few clinicians had concerns regarding cervical marginal adaptation and restoration failure. However, the routine clinical use of DME varied among practitioners across different regions and practice settings. These findings indicate variability in clinicians’ perceptions and application of DME in restorative practice.

## Introduction

Management of deep subgingival margins remains a persistent clinical challenge in restorative dentistry. Lesions extending below the cemento-enamel junction complicate isolation, compromise adhesive bonding, and increase the risk of violating the supracrestal tissue attachment. These factors may negatively influence marginal adaptation, periodontal health, and long-term restoration survival [1,2]. Achieving predictable outcomes in such situations requires techniques that allow adequate access, moisture control, and biologically respectful margin placement.

Deep margin elevation (DME) has emerged as a restorative strategy to facilitate management of subgingival defects by relocating the cervical margin coronally using adhesive restorative materials. The concept was first described by Dietschi and Spreafico [3] and later refined and popularized by Magne and colleagues [4]. The procedure, also referred to as cervical margin relocation, proximal box elevation (PBE), open sandwich technique, and coronal margin relocation (CMR), involves placement of an adhesive composite restorative to elevate deep cervical margins to a supragingival or equigingival level, thereby improving access and bonding conditions [5–7].

DME is considered a promising restorative option to facilitate the treatment of advanced caries lesions with dentin/cementum margins located beneath the gingival tissues, provided adequate isolation technique, the use of appropriate materials, and careful handling are employed [8,9]. Under controlled clinical conditions, the technique may facilitate restoration of advanced carious lesions while maintaining periodontal stability [8]. Composite resin and resin-modified glass ionomer cement are the most commonly used materials for deep margin elevation. Composite is typically preferred when good isolation can be achieved, while resin-modified glass ionomer is often selected in areas where moisture control is more challenging [10].

Historically, surgical crown lengthening (SCL) and orthodontic extrusion have been the principal approaches for managing subgingival margins prior to definitive restoration [10,11].Surgical crown lengthening often requires osseous recontouring to establish an adequate distance between the bone crest and restorative margin, which may result in attachment loss, root exposure, compromised esthetics, and altered crown–root ratios [12]. Orthodontic extrusion, while conservative, is associated with prolonged treatment duration and may introduce additional biomechanical and hygiene-related challenges [13]. In this context, DME has been proposed as a minimally invasive restorative alternative or adjunct in appropriately selected cases [14].

Although DME is increasingly discussed in contemporary restorative literature, its clinical adoption appears variable. Previous surveys conducted within individual countries have assessed practitioners’ awareness and reported use of DME, demonstrating heterogeneous levels of familiarity and implementation [15–17].

However, these studies were conducted on similar practices and did not evaluate variations in perceptions between clinical and academic practice setups across different practice environments. [15–17]. This study aimed to evaluate the level of awareness, perceptions, and clinical use of deep margin elevation for the management of subgingival restorative margins among practicing dentists from selected regions and different practice settings.

It further sought to determine how familiar dental practitioners are with deep margin elevation, assess its reported clinical use in subgingival restorative cases, and examine whether awareness and acceptance vary according to practice environment or professional background. In addition, the study explored current clinical preferences for alternative treatment approaches, particularly surgical crown lengthening and orthodontic extrusion, when managing deep subgingival margins. The first null hypothesis (H_01_) stated that no significant differences exist in awareness among practitioners with different practice settings and professional backgrounds. The second null hypothesis (H_02_) is that no significant differences in clinical acceptance across different practice settings and professionals.

## Methodology

### Study design and ethical approval

A cross-sectional questionnaire-based study was conducted to assess awareness and clinical acceptance of deep margin elevation (DME) among practicing dentists from selected regions, including the Middle East, the United States, Asia (India), Europe (UK) and practicing dentists in the fellows of the Academy of Dentistry International, stated as other regions. The regions selected were chosen for their accessible professional networks and practitioner groups available during the survey and were not intended to represent all geographic regions.

Ethical approval was obtained from the Virginia Commonwealth University Institutional Review Board (VCU IRB; approval no. HM20023469) on 15 December 2021. Participation was voluntary, and electronic informed consent was obtained from all respondents prior to completion of the survey. Although preregistration was not performed, all study objectives, survey items, and analyses were predefined before participant recruitment.

### Study population and timeline

Licensed dental practitioners working in academic institutions, private practices, or combined academic–private practice settings within the selected regions were eligible to participate. Dentists who declined participation or had difficulty completing the questionnaire in English were excluded. Data collection was conducted over a defined period from 25 January 2022–31 May 2022, during which the questionnaire was distributed, and responses were obtained

### Sample size calculation

Sample size estimation was based on the study null hypothesis, which stated that no significant differences exist in awareness or clinical acceptance of DME among practitioners across practice settings and professional backgrounds which could be evaluated with chi-squared tests. Based on a previously reported awareness level of 65% [15], the sample size was calculated using a 0.05 significance level and 80% power and determined that differences in awareness or perceptions and clinical use would be detectable if the difference in groups reached an odds ratio of 0.5 with a sample size of 270 if the group sizes were equal and 305 for a ratio of group sizes of 2:1. A target sample size of 350 respondents was therefore established to ensure adequate precision and allow subgroup comparisons relevant to the study hypothesis.

### Questionnaire development and validation

A structured, self-administered questionnaire was developed following review of the relevant literature on DME and subgingival restorative management. The instrument consisted of three sections: demographic and professional characteristics, awareness of DME, and clinical acceptance and use of DME in restorative practice. The final questionnaire included 20 closed-ended questions.

Content validity was evaluated by a panel of 15 experts in restorative dentistry and related disciplines who assessed clarity, relevance, and comprehensiveness. Revisions were made accordingly. A pilot evaluation was subsequently performed to assess clarity and reliability. Internal consistency analysis demonstrated acceptable reliability (Cronbach’s alpha = 0.82). Minor wording adjustments were made before final distribution.

### Data collection procedure

The validated questionnaire was distributed using convenience and snowball sampling through academic institutions, private practices, and professional networks across the included regions. Surveys were delivered in electronic and hard-copy formats, depending on participant accessibility. Electronic responses were collected and managed using Research Electronic Data Capture (REDCap) hosted at Virginia Commonwealth University, a secure web-based research data platform [18]. Two reminders were issued via email or telephone during the data collection period to improve response rates. All responses were anonymized to maintain confidentiality.

### Data analysis

Collected data were reviewed for completeness and consistency prior to analysis. Responses were summarized using descriptive statistics, including frequencies and percentages. Associations between awareness and clinical acceptance variables and respondent characteristics, such as practice setting and geographic region, were examined using chi-squared tests. Statistical analyses were performed using SAS Enterprise Guide version 8.3 (SAS Institute, Cary, NC, USA), with a significance level of p < 0.05.

## Results

### Respondent characteristics

A total of 349 dentists completed the survey and were included in the analysis. More than half of respondents practiced in the Middle East (n = 184, 53%), followed by the United States (n = 54, 15%) and Asia (India) (n = 48, 14%), with the remaining respondents from Europe (UK) (n = 11, 3%) and the fellows of the Academy of Dentistry International stated as other regions. (n = 49, 14%). Practice settings included purely private practice (n = 134, 39%), combined academic and private practice (n = 131, 38%), and purely academic practice (n = 79, 23%).

Additional training was reported in prosthodontics (n = 50, 14%), advanced education in general dentistry (n = 42, 12%), and operative dentistry (n = 43, 12%), while 29% reported no additional formal training. A detailed summary of respondent characteristics is presented in Table 1.

**Table 1:** Respondent characteristics.

Characteristic	n	%
**Practice setting**		
Purely academic/educational setting	79	23
Purely private dental practice	134	39
Combination of academic and private dental practice	131	38
Missing	3	1
**Country/region of practice**		
United States of America	54	15
Europe (UK)	11	3
Asia (India)	48	14
Middle East	184	53
Other (Fellows ADI)	49	14
Missing	3	1
**Years in practice**		
< 5 years	84	24
5–10 years	72	21
> 10 years	192	55
Missing	1	0
**Additional training**		
AEGD (Advanced Education in General Dentistry)	42	12
GPR (General Practice Residency)	29	8
Operative dentistry	43	12
Prosthodontics	50	14
Other	79	23
None	102	29
Missing	4	1

Regional participation was not evenly distributed, with greater representation from the Middle East compared with other surveyed regions.

### Associations with geographic region

Respondents were grouped into Middle East/Asia (n = 232, 67%) and United States/UK (n = 114, 33%). Practitioners from the Middle East and Asia were more likely to agree that DME improves tooth longevity (86% vs 76%; P = 0.029) and that DME performed under rubber dam isolation provides predictable results (87% vs 71%; P < 0.001).

Respondents from the United States and UK more frequently considered anterior teeth with minimal occlusal load to be suitable for predictable DME outcomes (54% vs 41%; P = 0.005). Differences in perceptions regarding DME use in patients with bruxism (P = 0.061) and preference for DME compared with orthodontic extrusion or surgical crown lengthening (P = 0.068) were not statistically significant. Table 1 & (S1 Fig)

Preferences for alternative treatments, such as orthodontic extrusion and surgical crown lengthening, were broadly similar across groups. Despite generally high awareness of DME, variations in reported clinical acceptance, material selection, and treatment preferences were observed across practice settings and geographic regions.

#### Awareness of deep margin elevation.

Most respondents were familiar with deep margin elevation (DME) or its related terminology (n = 268, 77%). Two hundred twenty-eight participants (66%) reported that predictable adhesive bonding to cervical or root dentin can be achieved in restorations with deep margins. The majority agreed that the gingival location of restoration margins influences restoration success by affecting marginal adaptation (n = 293, 85%).

Bitewing radiographs were most frequently selected for evaluation of subgingival marginal adaptation (n = 225, 65%), followed by intraoral periapical radiographs (n = 89, 26%), while panoramic radiographs were less frequently selected (n = 32, 9%). Most respondents identified 2 mm as the minimum biologic width required for restorative procedures (n = 201, 58%), and 96% (n = 331) agreed that evaluation of the existing biologic width is essential before selecting DME for subgingival lesions. Detailed awareness-related responses are presented in Table 2.

**Table 2:** Awareness of deep margin elevation.

Item / Response	n	%
**Can predictable adhesive bonding to cervical/root dentin be achieved in restorations with deep margins?**		
No	119	34
Yes	228	66
**Have you heard of DME (deep margin elevation/cervical margin relocation/proximal box elevation/open sandwich technique)?**		
No	79	23
Yes	268	77
**Does gingival location of restoration margins influence marginal adaptation and success?**		
No	53	15
Yes	293	85
**Best radiograph to evaluate subgingival marginal adaptation**		
Intraoral periapical (PA)	89	26
Bitewing (BW)	225	65
Orthopantomograph (OPG/Panoramic)	32	9
**Minimum biologic width required for restoration**		
1 mm	66	19
2 mm	201	58
3 mm	71	20
4 mm	9	3
**Evaluate existing biologic width before choosing DME**		
Disagree	14	4
Agree	331	96
**DME performed under a rubber dam will give predictable results**		
Disagree	63	18
Agree	280	82

### Clinical acceptance and practice patterns

Overall perceptions of DME were favorable. Most respondents agreed that DME may improve tooth longevity (n = 288, 83%) and increase the likelihood of restoration success (n = 287, 84%). However, 49% (n = 170) indicated concern that DME may lead to restoration failure due to inadequate cervical marginal adaptation.

When compared with alternative approaches, 79% (n = 271) preferred performing DME under adequate isolation with definitive restoration rather than orthodontic extrusion or surgical crown lengthening. Nevertheless, when favorable clinical conditions for surgical crown lengthening were present, 75% (n = 260) reported a preference for this surgical approach. In situations with an ideal crown–root ratio of 1:1.5, 54% (n = 188) preferred orthodontic extrusion.

Most respondents acknowledged potential complications associated with orthodontic extrusion and surgical crown lengthening, including root resorption, tooth fracture, attachment loss, and furcation exposure (83%–84%). For teeth requiring endodontic treatment, preferences regarding the timing of DME varied, with 42% favoring placement before endodontic therapy, 37% after, and 21% performing both procedures during a single visit.

Preferred levels of DME placement were nearly evenly divided between 2 mm supragingival (n = 163, 48%) and equigingival placement (n = 175, 52%). Composite (n = 139, 41%) and resin-modified glass ionomer cement (n = 141, 41%) were the most frequently selected materials for DME. Lithium disilicate CAD/CAM ceramic was the most commonly selected definitive restorative material placed over elevated margins (n = 101, 43%). Posterior teeth with normal occlusal loading were considered most suitable for predictable outcomes with DME (n = 252, 71%). These findings are summarized in Table 3.

**Table 3:** Clinical acceptance and practice patterns related to deep margin elevation.

Item / Response	n	%
**DME improves clinical longevity of the tooth**		
Disagree	61	17
Agree	288	83
**DME improves the success of the final restoration**		
Disagree	56	16
Agree	287	84
**DME may cause restoration failure due to poor cervical marginal adaptation/seal**		
Disagree	174	51
Agree	170	49
**Prefer orthodontic extrusion over DME when the ideal crown/root ratio exists**		
Disagree	158	46
Agree	188	54
**Complications of orthodontic extrusion may compromise prognosis**		
Disagree	55	16
Agree	290	84
**Prefer surgical crown lengthening (SCL) over DME when favorable conditions exist**		
Disagree	85	25
Agree	260	75
**SCL may lead to attachment loss or furcation exposure, affecting prognosis**		
Disagree	60	17
Agree	286	83
**Timing of DME in teeth requiring endodontic treatment**		
Before endodontic treatment	146	42
After endodontic treatment	128	37
Both in a single visit	73	21
**Prefer DME with good isolation and definitive restoration over extrusion/SCL**		
Disagree	74	21
Agree	271	79
**Level at which DME is performed**		
2 mm supragingival	163	48
Equi gingival	175	52
**Material of choice for DME**		
Composite	139	41
Conventional GIC	63	18
Resin-modified GIC	141	41
**Material of choice for definitive restoration over an elevated margin**		
Lithium disilicate CAD/CAM ceramic	101	43
Zirconia 3Y CAD/CAM	50	21
Zirconia 4Y CAD/CAM	35	15
Zirconia 5Y CAD/CAM	23	10
Any CAD/CAM ceramic	128	54
**Teeth where DME is predictably successful (multiple responses allowed)**		
Posterior teeth with normal masticatory load	252	71
Bruxers	71	20
Anterior teeth with minimal masticatory load	156	44

### Associations with practice setting

Several responses varied significantly according to practice setting (Table 4). Academic practitioners more frequently agreed that predictable adhesive bonding to cervical or root dentin can be achieved (77% vs 65% and 58%; P = 0.018). Differences were also observed in perceived minimum biologic width (P = 0.003), with academic respondents more likely to report 1 mm and those in private or mixed practice more likely to report 3 mm.

**Table 4:** Provider type associations.

Variable	Academic (n = 79) n (%)	Private practice (n = 134) n (%)	Combination (n = 131) n (%)	P value
Predictable adhesive bonding achievable	61 (77)	86 (65)	76 (58)	0.0182
Heard of DME technique	58 (74)	102 (76)	104 (80)	0.5989
Gingival location affects restoration success	65 (82)	113 (86)	110 (85)	0.8102
**Best radiograph for subgingival marginal adaptation**				<0.0001
Intraoral periapical	38 (48)	27 (20)	23 (18)	
Bitewing	25 (32)	103 (77)	93 (72)	
OPG/Panoramic	16 (20)	3 (2)	13 (10)	
**Minimum biologic width**				0.0028
1 mm	24 (31)	20 (15)	22 (17)	
2 mm	40 (51)	88 (66)	69 (53)	
3 mm	10 (13)	26 (19)	34 (26)	
4 mm	4 (5)	0 (0)	5 (4)	
Evaluate biologic width before DME (agree)	77 (99)	126 (95)	123 (95)	0.3458
DME improves clinical longevity (agree)	70 (89)	107 (80)	106 (81)	0.2372
DME improves restoration success (agree)	70 (90)	105 (80)	107 (84)	0.1578
DME may cause restoration failure (agree)	49 (63)	61 (46)	58 (45)	0.0282
Prefer orthodontic extrusion over DME (agree)	58 (75)	58 (44)	69 (53)	<0.0001
Orthodontic extrusion complications affect prognosis (agree)	66 (86)	111 (84)	108 (82)	0.8211
Prefer surgical crown lengthening (agree)	63 (81)	98 (73)	96 (75)	0.4502
SCL complications affect prognosis (agree)	66 (84)	108 (82)	107 (82)	0.9498
**Timing of DME with endodontic treatment**				0.2544
Before	33 (42)	51 (38)	59 (45)	
After	23 (29)	56 (42)	48 (37)	
Same visit	22 (28)	26 (19)	24 (18)	
Prefer DME over extrusion/SCL (agree)	70 (95)	95 (73)	102 (79)	0.017
**Level of DME**				0.0006
2 mm Supragingival	50 (68)	58 (44)	52 (40)	
Equi gingival	24 (32)	72 (55)	77 (60)	
**Material for DME**				0.3238
Composite	38 (49)	47 (36)	52 (40)	
Conventional GIC	15 (19)	23 (17)	25 (19)	
Resin-modified GIC	25 (32)	61 (46)	52 (40)	
**Definitive restoration material**				0.0045
Lithium disilicate CAD/CAM	35 (44)	28 (22)	37 (30)	
Zirconia 3Y	12 (15)	26 (20)	10 (8)	
Zirconia 4Y	9 (11)	11 (9)	15 (12)	
Zirconia 5Y	5 (6)	8 (6)	10 (8)	
Any CAD/CAM ceramic	18 (23)	55 (43)	53 (42)	
Rubber dam gives a predictable result (agree)	74 (97)	100 (76)	104 (80)	0.0003
**Teeth where DME was successful**				
Posterior teeth normal load	57 (71)	99 (74)	91 (69)	0.7247
Bruxers	16 (20)	29 (22)	26 (20)	0.9326
Anterior minimal load	25 (31)	68 (51)	61 (47)	0.0223

Academic respondents were more likely to agree that DME may lead to restoration failure due to cervical marginal adaptation issues (63% vs 45%–46%; P = 0.028) and to prefer orthodontic extrusion when an ideal crown–root ratio existed (75% vs 44%–53%; P < 0.001). Preference for performing DME under adequate isolation with definitive restoration was also higher among academic respondents (95% vs 73%–79%; P = 0.017).

Differences were also observed in preferred DME level (P = 0.001), restorative material selection (P = 0.005), and perceptions regarding predictability of rubber dam isolation (P < 0.001). Academic respondents were less likely to consider anterior teeth with minimal occlusal load as predictably successful for DME (31% vs 47%–50%; P = 0.022) (S2 Fig).

Academic practitioners tended to report slightly higher agreement that deep margin elevation improves both tooth longevity and restoration success compared with private and mixed practitioners, although these differences were not statistically significant. A notable difference was seen in treatment preference, where academic clinicians were more likely to favor orthodontic extrusion when an ideal crown–root ratio exists, while overall perceptions of complications and surgical crown lengthening remained similar across groups.

These differences indicate that familiarity with the technique does not necessarily translate into uniform clinical adoption and support the need for broader evaluation of current perceptions and use of DME across diverse practice environments.

## Discussion

This study evaluated awareness and clinical acceptance of deep margin elevation (DME) among practicing dentists from different practice environments and geographic regions. The findings indicate that although familiarity with DME is relatively high, its clinical acceptance and reported use remain inconsistent. These variations highlight a persistent gap between theoretical knowledge of the technique and its integration into routine restorative decision-making. Such discrepancies help explain why further evaluation of practitioner perceptions and practice patterns was necessary.

Most previously published surveys assessing awareness, knowledge, and use of DME have been conducted within single institutions or single countries, limiting the ability to compare practice patterns across different clinical environments and training backgrounds [15–17]. Regional [1,2] studies from India and other localized settings have reported increasing awareness of DME but variable clinical adoption [15,19]. The present investigation expands on this work by including practitioners from multiple regions and practice settings and by examining differences between academic and private practice environments. This broader sampling provides additional insight into how training background and practice context may influence decision-making related to management of subgingival restorative margins. Awareness of DME among respondents was high and consistent with previous regional reports [15,19]. Differences between academic and private practitioners suggest that exposure to contemporary adhesive techniques and access to continuing education may influence confidence in the clinical application of DME. These findings reinforce the importance of assessing not only knowledge of emerging restorative techniques but also their perceived feasibility in daily practice. Concerns regarding the predictability of adhesive bonding to cervical or root dentin were more frequently reported among practitioners outside academic settings. Isolation is the key factor in the success of DME, and a rubber dam is a must [20]. Contemporary adhesive systems have demonstrated the potential for durable bonding to deep cervical dentin when strict isolation and appropriate material protocols are followed [21]. Sixth-generation bonding agents achieve a predictable bond to cervical dentin because they are less technique sensitive, have a short application time and do not affect whether dentin is wet or dry [22]. Infiltration of resin occurs simultaneously with the self-etching process. Selective etching for enamel is recommended when present [23]. Amongst the materials selected for restoration, the short-fibre-reinforced flowable composite at the base is reported to produce a good marginal seal [24]. Additionally, using a bulk fill composite to restore the tooth with longer curing times, 40 seconds to ensure complete curing, supports favorable outcomes [25]. Nonetheless, the technique remains sensitive to clinical execution, and variability in operator experience or clinical environment may affect practitioner confidence. Radiographic selection patterns observed in this study reflect established restorative principles. Bitewing radiographs were most frequently preferred for evaluating proximal margins and subgingival adaptation, consistent with their documented diagnostic accuracy for assessment of posterior restorations [26]. Responses regarding required biologic width varied, although most respondents identified approximately 2 mm as the minimum dimension. There is a general consensus to evaluate biologic width before DME, suggesting a clear understanding of BW and its implications for the success of restoration [27]. Most respondents perceived DME as beneficial for improving restoration longevity and treatment outcomes, consistent with clinical reports describing DME as a conservative approach for managing deep cervical margins while preserving periodontal structures [4,7]. However, nearly half of respondents expressed concern regarding potential restoration failure related to cervical marginal adaptation. Multiple reasons from clinical procedure failure to choice of restoration can cause failure. When compared with alternative approaches, surgical crown lengthening and orthodontic extrusion continue to be widely considered. In our study, surgical crown lengthening (75%) was preferred over orthodontic extrusion (44%) [28]. Both procedures have documented limitations, including attachment loss, root exposure, esthetic compromise, and extended treatment time [14,28]. The continued preference for surgical crown lengthening under favorable conditions suggests that traditional surgical approaches remain deeply integrated into clinical decision-making despite growing interest in minimally invasive restorative strategies. Variability in responses regarding timing of DME in relation to endodontic treatment and preferred level of margin elevation reflects the absence of universally accepted clinical protocols. Previous clinical reports suggest that performing DME prior to definitive restoration may improve isolation and facilitate restorative procedures in selected cases [7,29]. Similarly, equigingival or slightly supragingival margin relocation has been recommended to optimize bonding and periodontal health [30]. Material selection patterns in the present study, favoring composite resin and resin-modified glass ionomer cement, align with existing literature describing both materials as suitable for margin elevation when proper isolation and adhesive protocols are followed [31,32]. These findings suggest that material selection is generally consistent with contemporary restorative recommendations.

The aim of the present study was to provide additional observational data regarding current perceptions and reported clinical use of DME among surveyed practitioners from different practice settings. The findings align with previously published reports while illustrating variability in treatment preferences and clinical decision making among the respondents.

Because statistically significant differences were identified in awareness and clinical acceptance measures across practitioner groups and practice environments, both null hypotheses were rejected.

### Limitations of the study

The sample was obtained through convenience and snowball sampling and was restricted to selected regions; therefore, findings should be interpreted as representative of the surveyed population rather than global practice. The study is self-reported and may introduce bias or overestimate clinical proficiency. A cross-sectional study captures data at a single point in time and does not account for cumulative experience. Thirdly, the data were collected across multiple centers, and the majority of respondents were from the Middle East, the USA, the UK, India and others; hence, the findings cannot be generalized to other regions. The use of an English-only instrument may have introduced selection bias by limiting participation. The specialist category was not a major one, leading to an imbalance in clinical perspectives, particularly regarding biologic width and periodontal considerations. Additional multicenter studies with larger and more geographically diverse samples would provide more generalizable data and allow deeper analysis of factors influencing clinical adoption of DME.

## Conclusion

The respondents demonstrated familiarity with DME and considered it a clinically acceptable approach when appropriate isolation could be achieved.

Awareness and clinical acceptance of DME varied according to practice setting, particularly in regard to adhesive predictability, biologic width assessment, restorative material selection, and preference for alternative treatment approaches.

Academic practitioners demonstrated greater caution with a stronger preference for DME under appropriate isolation.

Surgical crown lengthening and orthodontic extrusion remained commonly considered alternative treatment options, with treatment preferences differing amongst practitioners’ backgrounds.

DME approach reported varied among practitioners based on their experience and practice within the surveyed population.

## Supporting information

S1 Fig Region-wise distribution in the DME technique. (TIF)

S2 Fig Variation across practice type. (TIF)
